# RefDNN: a reference drug based neural network for more accurate prediction of anticancer drug resistance

**DOI:** 10.1038/s41598-020-58821-x

**Published:** 2020-02-05

**Authors:** Jonghwan Choi, Sanghyun Park, Jaegyoon Ahn

**Affiliations:** 1 Department of Computer Science, Yonsei University, Seoul, South Korea; 2 Department of Computer Science & Engineering, Incheon National University, Incheon, South Korea

**Keywords:** Computational models, Machine learning, Virtual drug screening

## Abstract

Cancer is one of the most difficult diseases to treat owing to the drug resistance of tumour cells. Recent studies have revealed that drug responses are closely associated with genomic alterations in cancer cells. Numerous state-of-the-art machine learning models have been developed for prediction of drug responses using various genomic data and diverse drug molecular information, but those methods are ineffective to predict drug response to untrained drugs and gene expression patterns, which is known as the cold-start problem. In this study, we present a novel deep neural network model, termed RefDNN, for improved prediction of drug resistance and identification of biomarkers related to drug response. RefDNN exploits a collection of drugs, called reference drugs, to learn representations for a high-dimensional gene expression vector and a molecular structure vector of a drug and predicts drug response labels using the reference drug-based representations. These calculations come from the observation that similar chemicals have similar effects. The proposed model not only outperformed existing computational prediction models in most comparative experiments, but also showed more robust prediction for untrained drugs and cancer types than traditional machine learning models. RefDNN exploits the ElasticNet regularization to deal with high-dimensional gene expression data, which allows identification of gene markers associated with drug resistance. Lastly, we described an application of RefDNN in exploring a new candidate drug for liver cancer. As the proposed model can guarantee good prediction of drug responses to untrained drugs for given gene expression patterns, it may be of potential benefit in drug repositioning and personalized medicine.

## Introduction

The development of computational models that can predict effective therapeutic strategies based on the genomic information of a cancer patient is a critical challenge in precision medicine^1,2^. Many effective anticancer drugs have been already developed and indicated for the treatment of cancer, but some patients with the same cancer types show different drug responses, such as drug resistance, owing to the tumour heterogeneity^3^. Since it has become evident that drug responses are strongly affected by genomic alterations in cancer^4^, numerous studies have been launched and have generated a large collection of drug response data for diverse cancer cell lines and various anticancer drugs. The major two studies to date are the Cancer Cell Line Encyclopaedia (CCLE) and Genomics of Drug Sensitivity in Cancer (GDSC) projects^4,5^. Enormous drug response datasets for almost 250 anticancer drugs across almost 1000 cancer cell lines were generated from these projects. In addition, those datasets are integrated with large genomic datasets for the cell lines, allowing the development of numerous computational models for predicting drug sensitivity based on genomic features of cell lines, as well as for searching for candidate compounds effective in drug-resistant tumours^6–9^.

Many computational models predict drug response using gene expression profiles and drug molecular data^9–11^. Wang *et al*. designed a machine learning model based on the observation that cell lines with similar gene expression patterns, or drugs with similar chemical properties, will exhibit similar responses^12^. The researchers thus developed a similarity-regularized matrix factorization (SRMF) method and predicted that rapamycin, an mTOR inhibitor, could be a new therapeutic agent for non-small cell lung cancer^11^. Zhang *et al*. proposed a graph-based approach using three network datasets, including drug-cell line response data, drug-target interactions, and protein-protein interactions. The authors integrated the three networks into one united network and applied an information-flow-based algorithm to the massive heterogeneous network for the prediction of drug responses^9^. These models showed good prediction performance, but it was not easy to obtain interpretable information related to drug mechanisms or pathways from the trained prediction model. To address this issue, Suphavilai *et al*. developed a model, termed Cancer Drug Response prediction using a Recommender System (CaDRReS), which could learn latent features for cell lines and drugs and demonstrated how to explore drug mechanisms and drug-pathway associations using the features^10^. These models showed good performance, but they are ineffective to make predictions about untrained new drugs or inexperienced new gene expression patterns, a factor referred to as the cold-start problem or new user/item problems^13^.

Deep neural networks (DNNs) are considered a powerful technology in diverse types of biological research^14^. For instance, DeepVariant, a universal caller using a DNN, showed superior performance compared to traditional genomic analysis tools^15^, and DCell presented a novel DNN-based approach for investigation of the molecular mechanisms underlying associations between genotype and phenotype^16^. Considering these results, the application of DNNs is expected to exhibit excellent performance in predicting therapeutic effects and investigating relationships between genomic features and drug resistance.

In the present study, we developed a novel Reference Drug-based Neural Network (RefDNN) model for effective prediction of anticancer drug response and identification of biomarkers contributing to drug resistance. RefDNN requires a pair consisting of the molecular structure data of a drug and gene expression profiles measured before exposure to the drug to predict the unknown drug response. RefDNN exploits a collection of drugs, called reference drugs, to compute the representations of the given gene expression vector and the drug structure data for effective prediction of drug response labels, sensitivity or resistance. These calculations, inspired by the observation that similar chemicals have similar effects, enable us to robustly predict untrained pairs of drugs and gene expression patterns. RefDNN consists of numerous ElasticNet models for computing representations of high-dimensional gene expression data and a DNN classifier for predicting drug response labels based on the representations. The proposed model is trained by two optimization algorithms, Follow-The-Regularized-Leader (FTRL)^17^ and Adam^18^, to effectively manage high-dimensional gene expression data and to investigate biomarkers for drug resistance. In addition, the Bayesian optimization approach is applied for efficient model tuning^19^. We used the GDSC and CCLE datasets as benchmark sets. The prediction performance of RefDNN was evaluated via nested cross-validation^20^. The proposed DNN model outperformed other machine learning classifiers and several state-of-the-art drug response prediction models in most comparative experiments. RefDNN also demonstrated more robust predictions of drug resistance for untrained drugs and cancer types than other machine learning models. After evaluating the prediction performance, we demonstrated how to identify genomic biomarkers associated with drug resistance to nilotinib using trained weights of ElasticNets contained in RefDNN. Lastly, we described an application of RefDNN in identifying a new candidate drug for the treatment of liver cancer.

## Results

### Description of benchmark datasets

In the present study, we used as benchmarks the GDSC and CCLE datasets, which were obtained from the Genomics of Drug Sensitivity in Cancer (https://www.cancerrxgene.org) and Cancer Cell Line Encyclopaedia websites (https://portals.broadinstitute.org/ccle), respectively. The GDSC dataset (version 17.3) provides the half maximal inhibitory concentration (IC_50_) values for 224,202 pairs that consists of 1,065 cancer cell lines and 251 drugs. We collected a cell line for which gene expression data was available and a drug with a Compound ID (CID) that could be searched in the PubChem database^21^ using the drug name. A CID was used to obtain a canonical simplified molecular-input line-entry system (SMILES) from the PubChem database. A continuous value of IC_50_ is converted to a binary value to deal with the task of drug resistance prediction as a binary classification problem^22^. Specifically, a pair with an IC_50_ value larger than the reported maximum screening concentration was categorized into the drug-resistance class and the others were assigned to the drug-sensitivity class. Our benchmark dataset from GDSC thus contained 120,606 resistance pairs and 69,430 sensitivity pairs consisting of 222 drugs and 983 cell lines. For the CCLE dataset, we exploited the Legacy Data in the website, which consists of 504 cell lines, 24 drugs, and 11,670 pairs. Since the CCLE dataset does not include the maximum screening concentration data, we used only 12 drugs shared with the GDSC. Applying the aforementioned procedure, we obtained 3,402 resistance pairs and 2,322 sensitivity pairs consisting of 12 drugs and 491 cell lines from the CCLE dataset. In the following experiments, all 222 drugs and the 12 drugs were used as reference drugs of the GDSC and CCLE datasets, respectively.

### Evaluation of prediction performance

**Figure 1:**
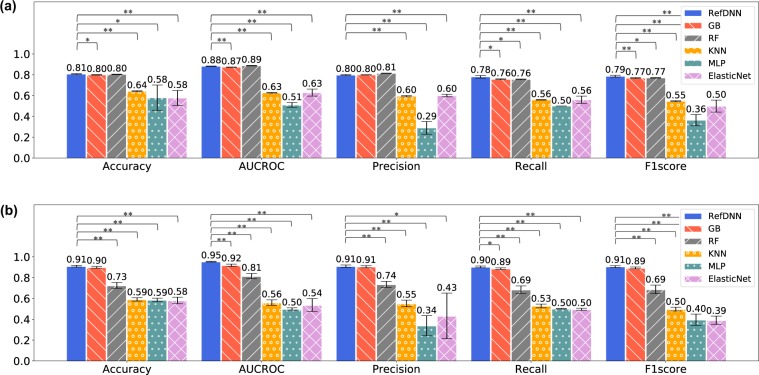
Evaluation of drug-resistance prediction performance of RefDNN on GDSC and CCLE datasets. (**a**) Results on GDSC dataset and (**b**) Results on CCLE dataset. The predictive power is computed by 5 metrics, accuracy, AUCROC, precision, recall, and F1score. A value on each bar is the average of accuracy values in the nested 5-fold cross-validation. An error bar represents the standard deviation in the cross validation. The significance of predictive performance differences between RefDNN and others was calculated using the Welch’s t-test and Benjamini-Hochberg procedure. Single and double asterisk symbols mean p < 0.05 and p < 0.01, respectively.

RefDNN can be superior to several machine learning models in the task of predicting drug resistance. We compared RefDNN to five classification models, ElasticNet, Gradient Boost (GB), K-Nearest Neighbour (KNN), Multilayer Perceptron (MLP), and Random Forest (RF) with respect to diverse metrics. Since the performance of baseline models depends on their hyperparameters, we exploited the nested cross-validation method that consists of an inner loop for model selection and an outer loop for model assessment to ensure fair comparison (Supplementary Fig. S1)^20^. Hyperparameter optimization in the inner loop was conducted using the Bayesian optimization approach^18^. The ranges of explored hyperparameters are enumerated in Supplementary Table S1. The prediction performance was computed by averaging the results of 5-fold cross-validation in the outer loop. We used five metrics, accuracy, precision, recall, f1score, and area under the receiver operating characteristic curve (AUCROC). Differences in those predictive performances between RefDNN and each baseline model was evaluated by the Welch’s t-test. The Benjamini-Hochberg procedure were used for multiple test correction. Figure 1 shows the results of comparative experiments conducted on the GDSC and CCLE datasets. RefDNN significantly outperformed the three traditional machine learning models including KNN, MLP, and ElasticNet classifiers in both datasets. In the cases of the two ensemble models GB and RF, RefDNN did not outperform them in some metrics, but showed sufficiently high predictive accuracy. The complete experimental results are shown in Supplementary Table S2.

**Figure 2:**
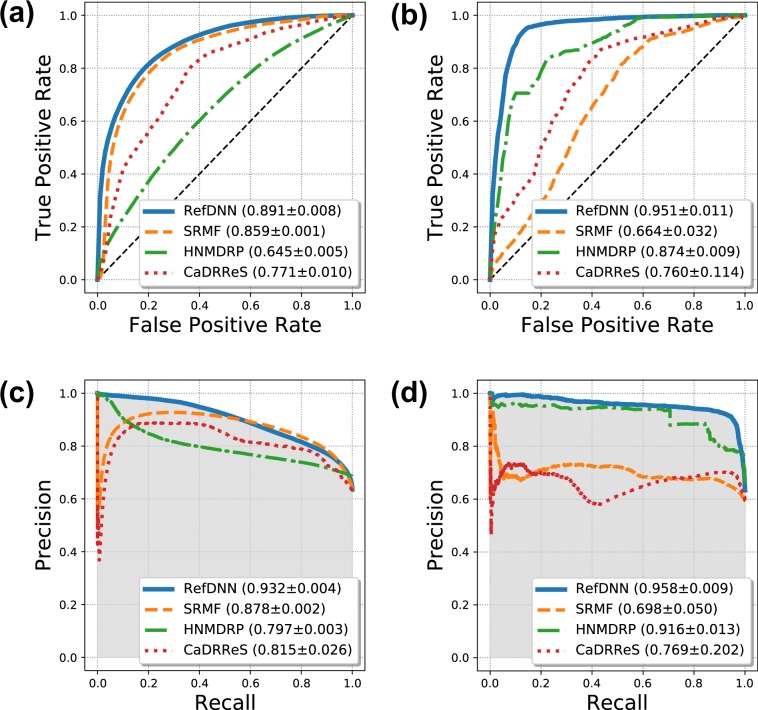
Comparison of the prediction performance of RefDNN with the state-of-the-art prediction models. (**a**) Receiver operating characteristic (ROC) curves of 5-fold cross-validation on GDSC dataset, (**b**) ROC curves on CCLE dataset, (**c**) Precision-Recall curve on GDSC dataset, and (**d**) Precision-Recall curve on CCLE dataset. The mean and standard deviation of accuracy of each model are shown in the plot legend. All p-values computed by Welch’s t-test were less than 0.05.

We also compared RefDNN to four state-of-the-art drug response prediction models, SRMF^11^, Heterogeneous Network-based Method for Drug-Response Prediction (HNMDRP)^9^, and CaRDDeS^10^ on the GDSC and CCLE datasets by means of the AUCROC and the area under the precision recall curve (AUCPR). On the GDSC dataset, the AUCROC and AUCPR values of RefDNN were 0.891 and 0.932, respectively (Fig. 2a,c). Those accuracies were significantly higher than that of those state-of-the-art prediction models up to 0.246 and 0.135, respectively (p < 0.01). The p-values were calculated via Welch’s t-test and corrected with the Benjamini-Hochberg procedure. We also confirmed that the AUCROC and AUCPR of RefDNN were higher than the others (0.071–0.287 and 0.042–0.260) on the CCLE dataset (Fig. 2b,d). As we expected, the proposed DNN model could achieve superior prediction accuracy to existing drug response prediction models not utilizing DNNs.

### Robust prediction for untrained drugs and cancer types

**Figure 3:**
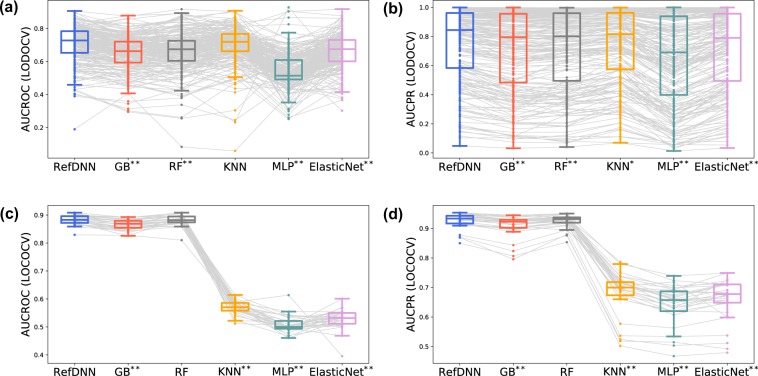
Prediction performance of RefDNN for untrained drugs and cancer types. (**a**) Box plots for AUCROC values of RefDNN and baseline models in LODOCV; (**b**) Box plots of AUCPR values in LODOCV; (**c**) Box plots for AUCROC values in LOCOCV; (**d**) Box plots of AUCPR values in LOCOCV. Differences in predictive performances between RefDNN and other machine learning models were assessed using the Wilcoxon signed-rank test and the Bonferroni correction. Single and double asterisk symbols mean p < 0.05 and p < 0.01, respectively.

RefDNN is a model that overcomes the new item problem and can robustly predict labels of response for drugs and cancer types on which the model was not previously trained. We evaluated the robustness through two experiments, leave-one-drug-out cross-validation (LODOCV) and leave-one-cancer-type-out cross-validation (LOCOCV) (Supplementary Fig. S2). In LODOCV, one drug and corresponding drug-cell line pairs to the drug are used as a test set and the remainder pairs are used as a training set. In a similar way, in LOCOCV, one cancer type and corresponding pairs are used for evaluation and the others are used for training a model. AUCROC and AUCPR values of RefDNN and the previously compared machine learning models are shown in Fig. 3. The complete results from the LODOCV and LOCOCV are provided in Supplementary Tables S3 and S4, respectively. We calculated the significance of performance differences using the Wilcoxon signed-rank test and the Bonferroni correction. RefDNN showed significantly higher AUCROC and AUCPR values than other machine learning models in most cases (Fig. 3). Although a significant difference was not observed between RefDNN and KNN in LODOCV, our model showed a high AUCROC value of over 70% on average. In LOCOCV, the performance gaps between RefDNN and RF were not statistically significant, but RefDNN was a good enough model to show accuracy of 0.85 or higher (Fig. 3c,d).

RefDNN showed robust predictive performance regardless of the type of cancer (Fig. 3c,d), but the deviation was large depending on the drug (Fig. 3a,b). To determine which type of drugs the RefDNN was strong and weak for, we grouped drugs by their target pathways and calculated the average accuracy of each group (Supplementary Table S5). The top five target pathways for which RefDNN showed an AUCPR of 0.85 or higher were ABL signalling, RTK signalling, cytoskeleton, hormone-related, and genome integrity pathways. On the other hand, RefDNN seems to be weak for drugs targeting apoptosis regulation (AUCROC of 0.725) and metabolism (AUCROC of 0.7).

### Effect of the number of reference drugs

The prediction power of RefDNN depends on the number of reference drugs because the number is an important hyperparameter for drug and cell line representations. We formed hypotheses that the higher the number of reference drugs, or the greater the difference in the molecular structures of reference drugs, the richer the representation spaces for drugs and cell lines would be. To validate the relationship between the predictive ability and the number of reference drugs, we conducted 5-fold cross-validation over the various numbers of references (50, 100, 150, and 200) (Supplementary Fig. S3). For each number of reference drugs, we selected 100 subsets of drugs randomly as reference drugs and evaluated three metrics, accuracy, AUCROC, and AUCPR based on the 100 reference drug sets. As we thought, as the number increased, the performance increased proportionally. The expected accuracy of RefDNN was confirmed to be 80% when the number of reference drugs was at least 150. To verify the second hypothesis, for each number, we explored the special subset in which drugs had maximally different molecular structures from each other among 100 subsets and checked the performance of that subset. We found that these maximally different subsets were able to ensure higher than average accuracy. Based on these results, it appears to be necessary to have at least 150 reference drugs with different molecular structures to guarantee good performance of RefDNN.

### Identification of biomarkers for drug resistance

**Figure 4:**
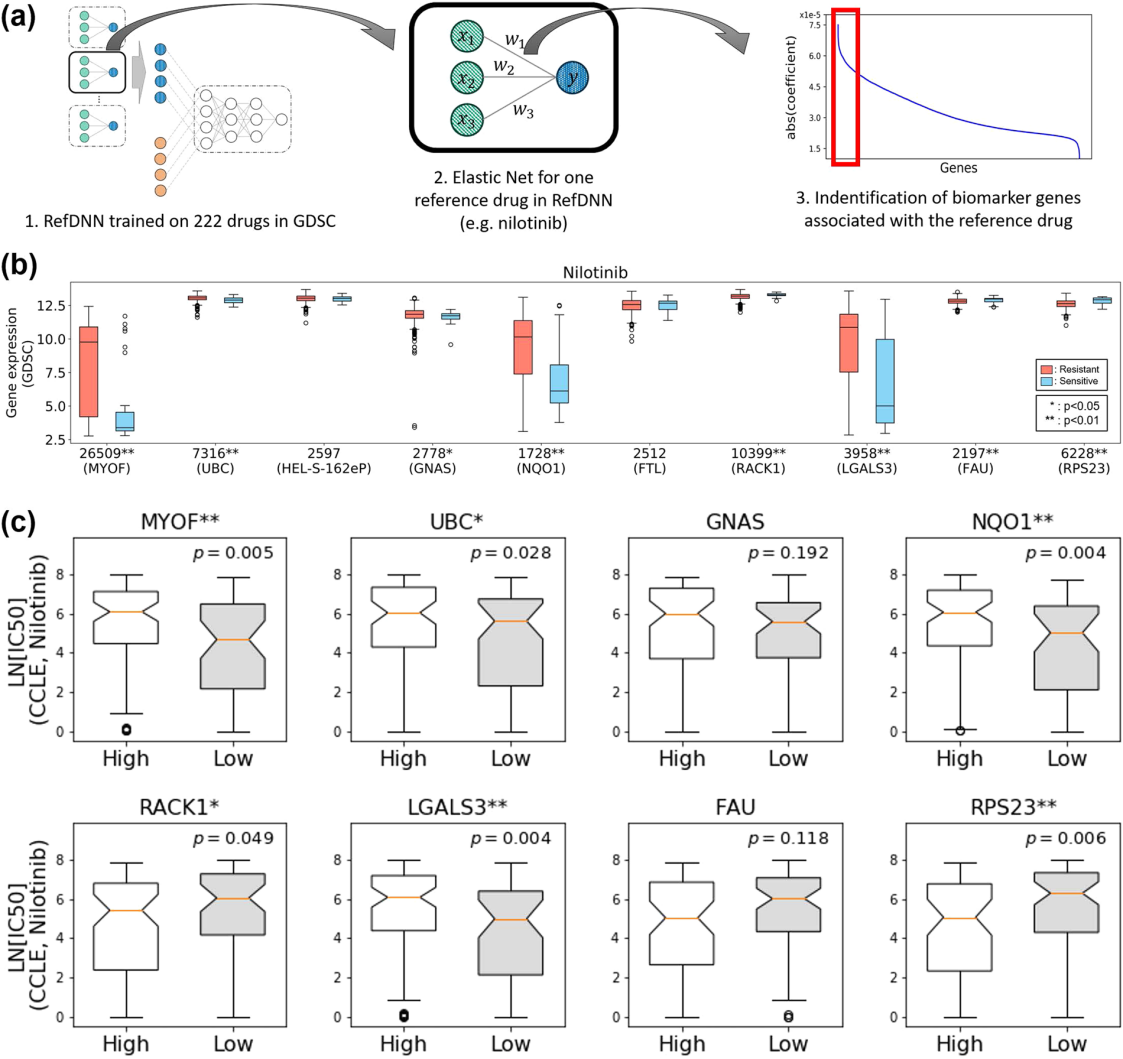
Identification and validation of biomarkers related to drug resistance. (**a**) Procedure of identification of biomarker candidates using RefDNN; (**b**) Top 10 candidate genes associated with nilotinib resistance and their expression patterns in cell lines resistant (red) and sensitive (blue) to nilotinib in GDSC dataset; (**c**) Validation of the relationship between IC_50_ of nilotinib and 8 differentially expressed candidate genes (MYOF, UBC, GNAS, NQO1, RACK1, FAU, LGALS3, and RPS23) in GDSC dataset using CCLE dataset. For each gene, a set of cell lines in CCLE dataset was divided into high and low expression groups by means of gene expression levels; All p-values were computed using the Mann-Whitney U test and corrected by the Benjamini-Hochberg procedure. Single and double asterisk symbols mean p < 0.05 and p < 0.01, respectively.

RefDNN can be utilized to identify a biomarker gene related to drug resistance owing to its unique neural network architecture combined with ElasticNet. RefDNN has multiple ElasticNet classifiers for computing representations of a high-dimensional gene expression vector. Each ElasticNet classifier is trained to learn drug resistance labels of each reference drug one-to-one and to reduce the prediction error of RefDNN. The trained ElasticNet models can prioritize genes depending on the degree to which genes are important in predicting drug resistance to corresponding reference drugs by l1 and l2 regularization^23^ (Fig. 4a). Unlike using the loss of ElasticNet alone, RefDNN updates the coefficients of ElasticNet using not only ElasticNet loss but also DNN loss. We expect that these multiple losses force ElasticNet models into identifying more effective biomarkers associated with drug resistance using the additional information backpropagated from DNN loss. To verify the power of biomarker identification of RefDNN, we collected the top 10 genes for each reference drug with the large absolute values of coefficients in corresponding ElasticNet model trained on the GDSC dataset as biomarker candidates (Supplementary Table S6) and investigated the 10 candidate genes associated with drug resistance to nilotinib, which is one of the shared drugs between the GDSC and CCLE datasets. Figure 4b shows the top 10 genes and their differentially expressed patterns between the groups resistant and sensitive to nilotinib in the GDSC datasets. Among the 10 candidates for drug resistance biomarkers, we identified 8 significantly differentially expressed genes (DEGs) between different nilotinib responses using the Mann-Whitney U test. To validate the utility of those 8 DEGs to determine drug resistance to nilotinib, we conducted additional differential expression analysis using the CCLE dataset. We divided CCLE samples into high and low expression groups by means of each DEG and evaluated the differences of natural logarithm values of IC_50_ between groups using the Mann-Whitney U test and the Benjamini-Hochberg procedure (Fig. 4c). Our results identified 6 genes, MYOF, UBC, NQO1, RACK1, LGALS3, and RPS23 that showed consistent differentially expressed patterns with GDSC in the CCLE dataset. Based on these results, high expression patterns of MYOF, UBC, NQO1, and LGALS3 and low expression patterns of RACK1 and RPS23 may be related to the causes of nilotinib resistance.

### Application for drug repositioning

**Figure 5:**
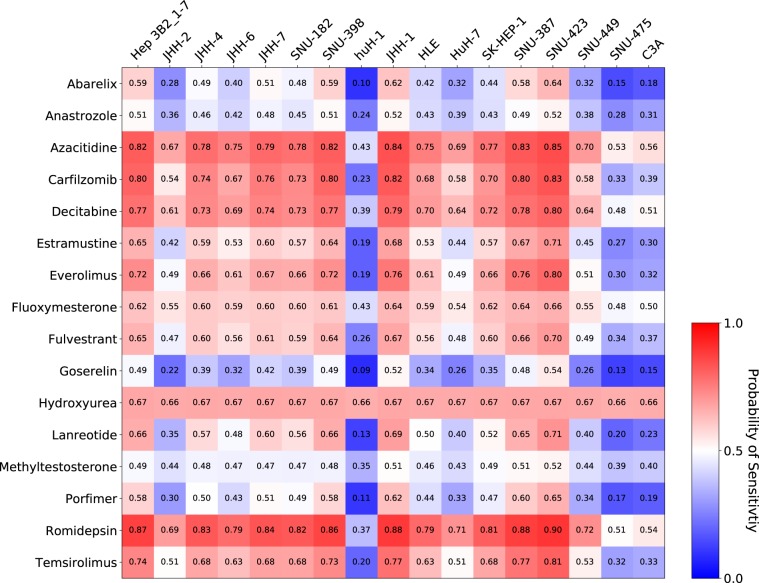
Prediction of drug sensitivity to FDA-approved anticancer agents of HCC cell lines. Rows and columns are anticancer drugs and HCC cell lines, respectively. The probability of sensitivity is computed by 1-probability of resistance. A score higher than 0.5 means that the corresponding row drug may be a novel repositioned drug for treatment of the corresponding column cell line.

RefDNN showed good predictive performance for untrained drugs in LODOCV experiments (Fig. 3a,b). Using RefDNN trained on the GDSC dataset, it is possible to explore a new candidate anticancer drug that was not tested in the GDSC project. Hepatocellular carcinoma (HCC) is one of the most prevalent cancers in the world and sorafenib, a multi-targeted kinase inhibitor, is the only currently approved chemotherapeutic agent for HCC^24^. Since sorafenib has only a modest effect, identifying an alternative drug for HCC remains a challenging task^25^. To address this issue, we collected data for 89 target-based drugs approved by the US Food and Drug Administration (FDA) from the study of Sun *et al*.^26^ and analysed the drug sensitivity of 37 agents that were not tested in the GDSC project (Supplementary Tables S7). In this experiment, we considered a drug to which a cell line is not resistant as a sensitive and effective drug to the cell line. Using the dataset of solid cancer cell lines in the GDSC, we predicted whether the 37 anticancer drugs could be effective in 17 HCC cell lines of the GDSC. Figure 5 shows 16 target-based drugs that were predicted to be sensitive in at least one HCC cell line. Among the identified drugs, 8 drugs (azacitidine^27^, carfilzomib^28^, everolimus^29^, fulvestrant^30^, goserelin^31^, lanreotide^32^, romidepsin^33^, and temsirolimus^34^) have been studied for treatment of HCC. An *in vitro* study reported that azacitidine, a nucleoside metabolic inhibitor, induced cell death in HuH7 cell lines and the cytotoxicity could be increased by drug combination with alendronate^27^. A recent study found that carfilzomib, a proteasome inhibitor indicated for treatment of multiple myeloma, could induce apoptosis in Hep3B cell lines and improve the drug sensitivity to sorafenib in HCC^28^. Romidepsin is a histone deacetylase inhibitor indicated for treatment of refractory peripheral cutaneous T-cell lymphoma. An *in vivo* study observed that romidepsin was involved in G2/M phase cell cycle arrest and promoted apoptosis in HuH7 cell lines^33^. These experimental results support our claim that RefDNN can be useful in drug repositioning and the other 8 drugs (abarelix, anastrozole, decitabine, estramustine, fuloxymesterone, hydroxyurea, methyltestosterone, porfimer) may be novel therapeutic agents for liver cancer.

## Discussion

In the present study, we propose a novel DNN model, termed RefDNN, for the accurate prediction of anticancer drug responses based on gene expression profiles and chemical structure information. The proposed prediction model showed higher predictive accuracy, equal or greater than that of the existing computational models (Figs. 1 and 2). We also confirmed that RefDNN was able to predict drug resistance robustly for untrained drugs and cancer types (Fig. 3). Our DNN model has a special architecture containing multiple ElasticNet classifiers, which allows us to identify genomic biomarkers contributing to drug resistance (Fig. 4). These results taken together suggest that the proposed model can be useful in numerous areas of therapeutic research, such as drug repositioning and personalized medicine.

RefDNN has five hyperparameters affecting prediction performance (Supplementary Tables S1), and these can be automatically tuned using the Bayesian optimization method. However, the optimization method does not always find desired optimal values, because the surrogate model of Bayesian optimization is sensitive to its parameters, such as the acquisition functions and the restricted range of each hyperparameter. In the present study, the configurations of the surrogate model were determined heuristically. A hyperparameter tuning job should be performed with a sufficiently large number of experiments for elevating the performance of RefDNN.

We demonstrated that the proposed model was able to overcome the cold-start problem and make good predictions for new drug and new cancer type data using reference drug-based representation method. Owing to its distinguishing features from existing models, RefDNN can be useful in a variety of tasks for the development of new target therapies. However, the proposed model has a limitation that it cannot be used for predicting protein-based therapies such as immunotherapy because their canonical SMILES are too long to compute finger-prints using PaDEL. In the future, we therefore plan to develop an upgraded model that can also predict the responses of those macromolecular drugs.

## Methods

### Pre-processing of gene expression data

Gene expression data from both the GDSC and CCLE were normalized using Robust Multi-array Average^35^ and the probe IDs were mapped to Entrez Gene IDs^36^ via mapping files GPL13667 and GPL15308 downloaded from the Gene Expression Omnibus database^37^, resulting in 17,780 and 18,926 genes from the GDSC and CCLE, respectively.

### Drug molecular structure similarity profile

RefDNN exploits structure similarity profiles (SSPs) based on user-defined reference drugs, instead of plain molecular fingerprints data, to reflect the fact that similar drugs have similar drug responses^12^. An SSP is a vector containing a number of Tanimoto coefficient values computed through comparison with binary fingerprints of reference drugs. In this study, a fingerprint of a drug was defined as a binary vector of length 3,072 consisting of Fingerprinter, ExtendedFingerprinter, and GraphOnlyFingerPrinter computed by PaDEL descriptor (version 2.21)^38^. For *M* reference drugs, the SSP vector ${{\boldsymbol{s}}}_{i}$ of $i$-th drug is computed by:

1 $${{\boldsymbol{s}}}_{i}=({\bf{T}}{\bf{c}}({{\boldsymbol{d}}}_{i},{{\boldsymbol{r}}}_{1}),\,{\bf{T}}{\bf{c}}({{\boldsymbol{d}}}_{i},{{\boldsymbol{r}}}_{2}),\,\ldots \,,\,{\bf{T}}{\bf{c}}({{\boldsymbol{d}}}_{i},{{\boldsymbol{r}}}_{M}))$$

In Eq. (1), ${{\boldsymbol{d}}}_{i}$ is the fingerprint of *i*-th drug, ${{\boldsymbol{r}}}_{j}$ is the fingerprint of *j*-th reference drug, and **Tc** is the Tanimoto coefficient quantifying the ratio of the number of features shared by two drugs to the number of their unique features in their molecular fingerprints^39^. Since the Tanimoto value lies on the closed unit interval of the real line ($0\le {\bf{T}}{\bf{c}}({{\boldsymbol{d}}}_{i},{{\boldsymbol{r}}}_{j})\le 1$), an SSP vector is used for weighting information from reference drugs for a target drug.

### RefDNN

**Figure 6:**
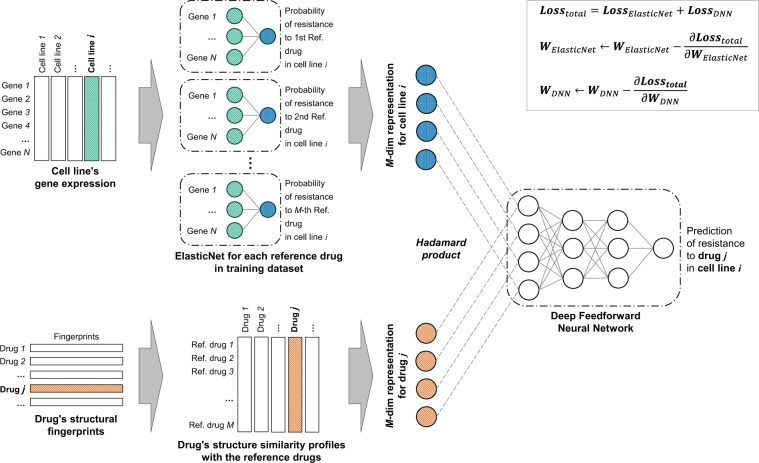
Overview of RefDNN. RefDNN consists of multiple ElasticNet classifiers trained by predicting the drug resistance labels of an input cell line to *M* reference drugs for generating a representation of gene expression data and a deep feedforward neural network classifier trained by taking two data representations of drug fingerprint data and cell line’s gene expression data and predicting the resistance of an input drug in the cell line. The drug representation is a drug structure similarity profile computed by the Tanimoto coefficient between fingerprints of an input and reference drugs. Whole weights of ElasticNet and DNN classifiers in RefDNN are updated by the gradient of total loss defined by the sum of ElasticNet loss and DNN loss.

RefDNN consists of one deep feedforward neural network (DNN) classifier and numerous ElasticNet logistic regressors (Fig. 6). For a given cell line, each ElasticNet model first predicts whether the cell line is resistant to each reference drug or not. A DNN classifier integrates the results of those ElasticNet models using an SSP vector of a target drug and then decides whether the cell line is resistant to the target drug or not. This architecture is based on an idea derived from the observation that structurally similar drugs tend to exhibit similar drug responses.

To describe mathematically the proposed model, we define some notations. Let $D={\{({{\boldsymbol{x}}}_{i},{{\boldsymbol{d}}}_{i},{y}_{i})\}}_{i=1}^{N}$ be a training dataset where ***x***_*i*_, ***d***_*i*_, and ${y}_{i}$ are a gene expression vector of an input cell-line, a fingerprint of a target drug, and binary response label for the *i*-th tuple. For *M* reference drugs, a collection of ElasticNet classifiers computes a probability vector ***z***_*i*_ with *M* lengths.

2 $${{\boldsymbol{z}}}_{i}=(\sigma ({{\boldsymbol{w}}}_{1}^{T}{{\boldsymbol{x}}}_{i}),\,\sigma ({{\boldsymbol{w}}}_{2}^{T}{{\boldsymbol{x}}}_{i}),\ldots ,\sigma ({{\boldsymbol{w}}}_{M}^{T}{{\boldsymbol{x}}}_{i}))$$

In Eq. (2), ***w***_*j*_ is the probability of the input cell line represented by ***x***_*i*_ is resistant to the *j*-th reference drug and $\sigma (x)$ is the sigmoid function $1/(1+{e}^{-x})$. A bias term is omitted for the convenience of description.

The probability vector ${{\boldsymbol{z}}}_{i}$ is weighted by an SSP vector ***s***_*i*_ of a target drug ***d***_*i*_ and used as an input vector of a DNN classifier that makes a final decision whether the input cell-line ***x***_*i*_ is resistant to the target drug ***d***_*i*_. The weighted probability vector ${\hat{{\boldsymbol{z}}}}_{i}$ is computed by the element-wise vector multiplication, called the Hadamard product.

3 $${\hat{{\boldsymbol{z}}}}_{i}={{\boldsymbol{z}}}_{i}\odot {{\boldsymbol{s}}}_{i}={(\sigma ({{\boldsymbol{w}}}_{j}^{T}{{\boldsymbol{x}}}_{i})\times {\bf{T}}{\bf{c}}({{\boldsymbol{d}}}_{i},{{\boldsymbol{r}}}_{j}))}_{j=1}^{M}$$

A feedforward neural network of RefDNN consists of three layers including two hidden layers ${{\boldsymbol{h}}}_{i}^{(1)},\,{{\boldsymbol{h}}}_{i}^{(2)}$ and one output layer ${\hat{y}}_{i}$. The two hidden layers contain equal numbers of units and perform a linear transformation, batch normalization *BN*, and nonlinear activation sequentially. Since the number of hidden units is one of the hyperparameters of RefDNN, an optimal value will be chosen automatically by the Bayesian optimization algorithm.

4 $${{\boldsymbol{h}}}_{i}^{(1)}=\sigma (BN({{\boldsymbol{W}}}_{1}^{T}{\hat{{\boldsymbol{z}}}}_{i}))$$

5 $${{\boldsymbol{h}}}_{i}^{(2)}=\sigma (BN({{\boldsymbol{W}}}_{2}^{T}{{\boldsymbol{h}}}_{i}^{(1)}))$$

The final output layer ${\hat{y}}_{i}$ has linear transformation and nonlinear activation and calculates the probability of drug resistance.

6 $${\hat{y}}_{i}=\sigma ({{\boldsymbol{w}}}_{output}^{T}{{\boldsymbol{h}}}_{i}^{(2)})$$

A loss of RefDNN is defined by the sum of losses of multiple ElasticNet models and loss of DNN classifier. A loss of DNN is defined by binary cross entropy and is always larger than zero.

7 $$Los{s}_{DNN}=-\,\mathop{\sum }\limits_{i=1}^{N}L({y}_{i},{\hat{y}}_{i})$$

In the Eq. (7), $L({y}_{i},{\hat{y}}_{i})={y}_{i}\,\log \,{\hat{y}}_{i}+(1-{y}_{i})\log (1-{\hat{y}}_{i})$ is a binary cross entropy.

On the other hands, a loss of ElasticNet can be zero depending on a given target drug ${{\boldsymbol{d}}}_{i}$. If a target drug is one of *M* reference drugs, then a loss of ElasticNet is computed in the ElasticNet model for the reference drug by binary cross entropy. If a target drug is not any of the reference drugs, then a loss of ElasticNet will be zero.

8 $$Los{s}_{ElasticNet}=-\,\mathop{\sum }\limits_{i=1}^{N}\mathop{\sum }\limits_{j=1}^{M}{1}_{{{\boldsymbol{d}}}_{i}={{\boldsymbol{r}}}_{j}}L({y}_{i},{z}_{ij})$$

In the Eq. (8), ${z}_{ij}=\sigma ({{\boldsymbol{w}}}_{j}^{T}{{\boldsymbol{x}}}_{i})$ defined in the Eq. (2) and ${1}_{{{\boldsymbol{d}}}_{i}={{\boldsymbol{r}}}_{j}}$ is an indicator function that has 1 if a target drug ${{\boldsymbol{d}}}_{i}$ is equal to the *j*-th reference drug ${{\boldsymbol{r}}}_{j}$ otherwise 0.

Training the ElasticNet and DNN classifiers is performed by two gradient-based optimization algorithms, FTRL and Adam optimizers. As a general gradient-based optimizer fails to produce a zero coefficient for weights, the FTRL optimizer was developed to overcome it^17^. Using the FTRL optimization, we can deal with the high-dimensional gene expression data and investigate the association between gene expression levels and drug resistance. Weight vectors of *M* ElasticNet classifiers ${\{{{\boldsymbol{w}}}_{j}\}}_{j=1}^{M}$ are optimized by FTRL and weight matrices of DNN classifier ${{\boldsymbol{W}}}_{1}$, ${{\boldsymbol{W}}}_{2}$, and ${{\boldsymbol{w}}}_{output}$ are updated via the Adam optimizer for rapid convergence^18^.

All weights in RefDNN are initialized by Glorot uniform initializer^40^. To prevent overfitting, we apportion a training set into training and validation sets with an 80–20 split and utilize early stopping^41^.

RefDNN is implemented by Python3 and Tensorflow1.12. The source code is freely available on GitHub at https://github.com/mathcom/RefDNN.

### Bayesian optimization and nested cross-validation

RefDNN has five hyperparameters including numbers of units of hidden layers of the DNN classifier, learning rates for the FTRL and Adam optimizers, and l1 and l2 regularization strength of the FTRL optimizer. To search the optimal values of these parameters, we exploited the Bayesian optimization implemented in scikit-optimize (https://scikit-optimize.github.io/). We defined an objective function for hyperparameter optimization by the prediction accuracy of 3-fold cross-validation and specified the range of each hyperparameter in Supplementary Table S1. In our hyperparameter tuning job, the acquisition and covariance functions were set as Expected Improvement (EI) with ξ = 0.01 and Matern 5/2 kernel, respectively^19,42^.

We allowed the Bayesian search algorithm to explore 20 combinations of the five hyperparameters of RefDNN and to select the best combination through the nested k-fold cross-validation. The nested k-fold cross-validation consists of two cross-validation loops, an outer loop for model assessment and an inner loop for model parameter selection. The nested k-fold cross validation splits the whole dataset into training and test sets via k-fold cross-validation for evaluating test accuracy, and another k-fold cross-validation was applied to the training dataset to evaluate validation accuracy and determine optimal parameter values (Supplementary Fig. S1).

## Supplementary information

Supplementary tables.

Supplementary figures.
