# Supplementary material for: RefDNN: a reference drug based neural network for more accurate prediction of anticancer drug resistance
Source: Sci Rep. 2020 Feb 5;10:1861. doi: 10.1038/s41598-020-58821-x (PMC7002431; doi:10.1038/s41598-020-58821-x)
Supplement: Supplementary file 2 — Supplementary figures. [file 41598_2020_58821_MOESM2_ESM.docx]

**RefDNN: a reference drug based neural network for more accurate prediction of anticancer drug resistance**

Jonghwan Choi, Sanghyun Park, and Jaegyoon Ahn

Supplementary Figures

**Index**

[**Supplementary Fig. S1.** 2](#_Toc5154428)

[**Supplementary Fig. S2.** 3](#_Toc5154429)

[**Supplementary Fig. S3.** 3](#_Toc5154430)


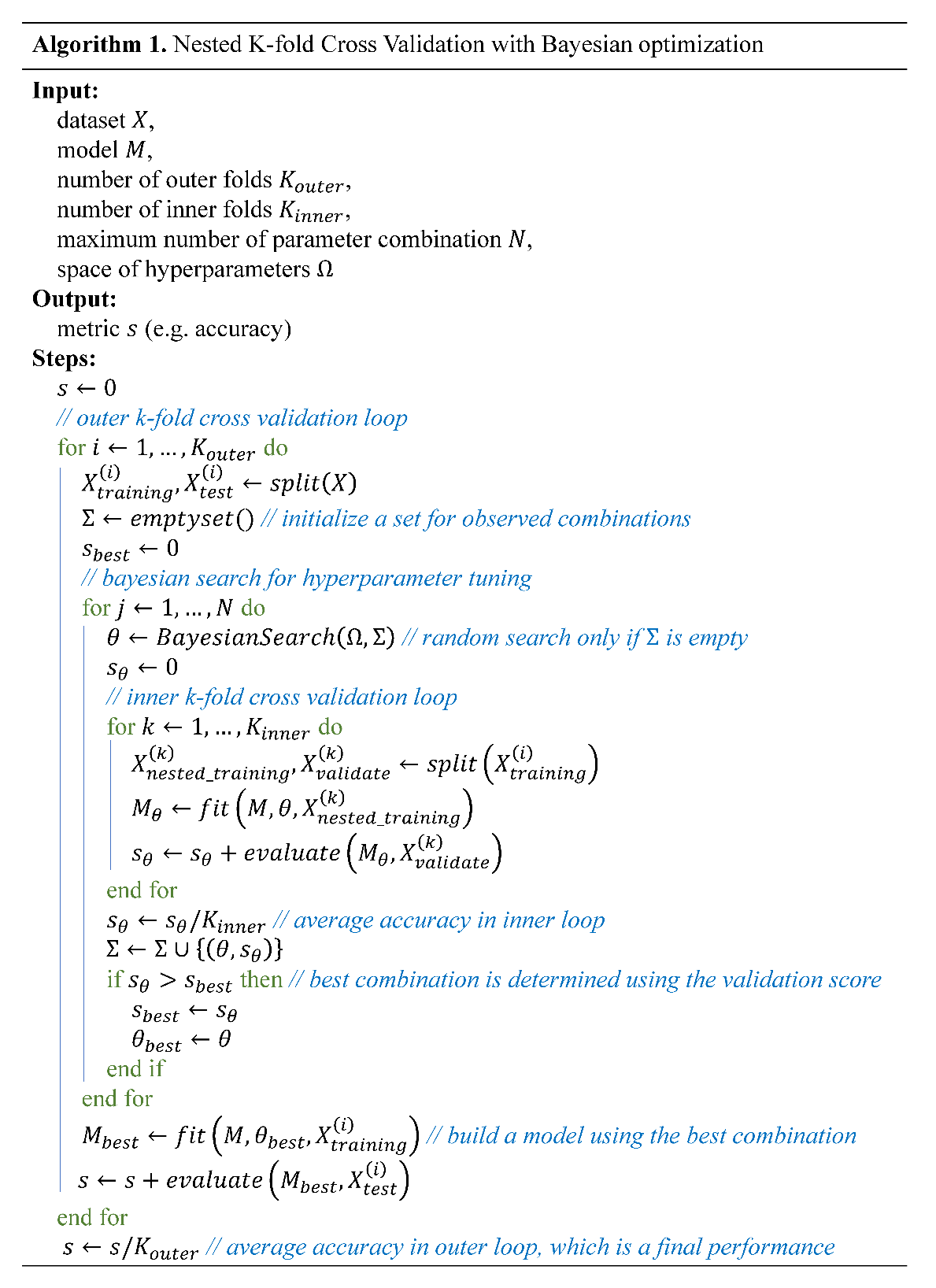


**Supplementary Fig. S1. Nested k-fold cross-validation with Bayesian optimization.** The nested k-fold cross-validation consists of an inner k-fold cross-validation loop for model selection and an outer k-fold cross validation loop for model assessment. The *BayesianSearch* function searches for a candidate of the best parameters based on previously observed combinations of parameter values. This search is repeated *N* times and the best combination of parameters is selected among the *N* observations by comparing the average of validation accuracy computed in the inner cross-validation loop. Using the best combination, a model is trained on the training set and evaluated by the test set.


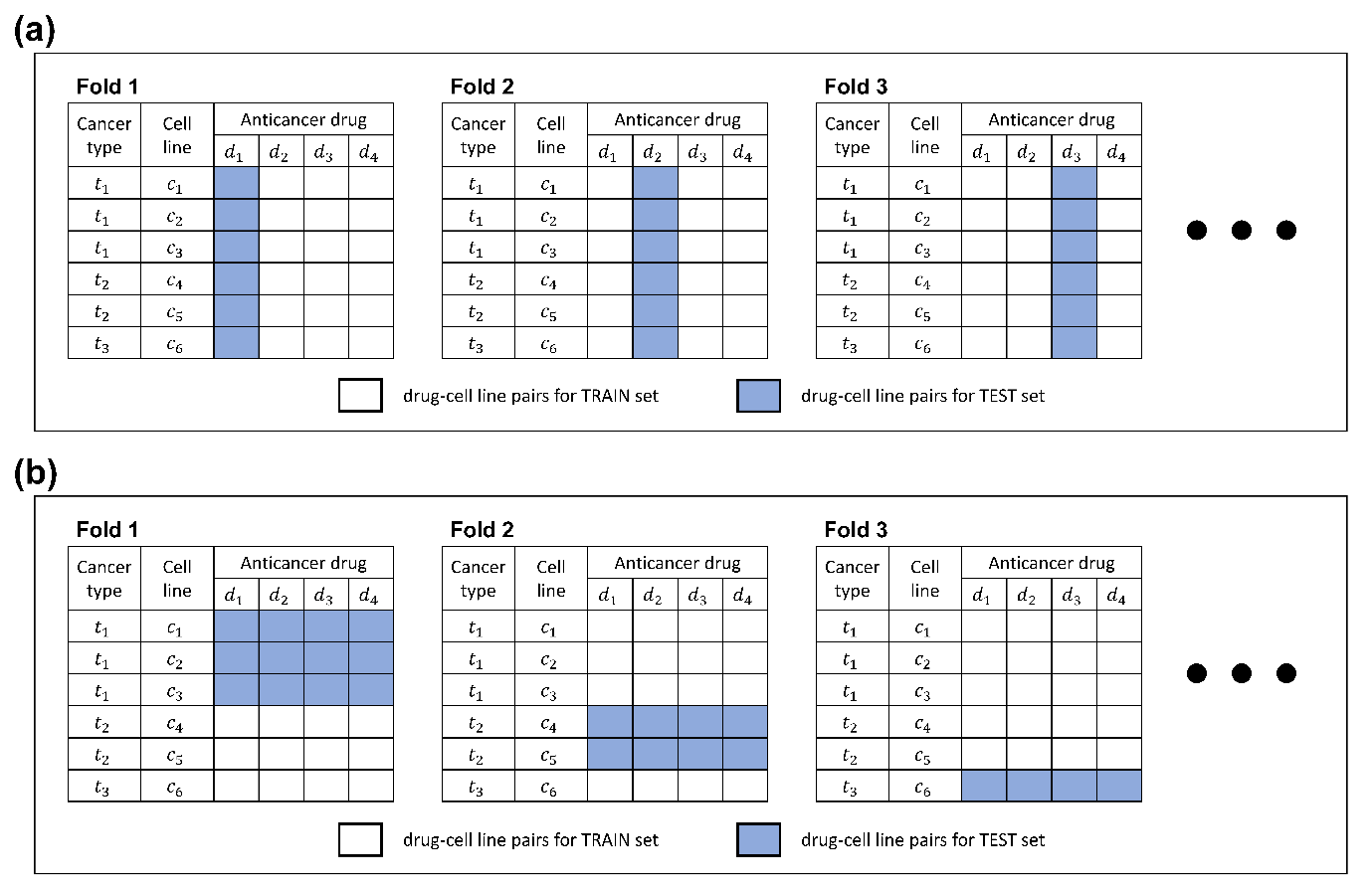


**Supplementary Fig. S2. Two leave-one-out cross-validation methods for evaluating robustness of prediction.** (a) Leave-one-drug-out cross-validation (LODOCV) and (b) Leave-one-cancer-type-out cross-validation (LOCOCV). These cross-validation experiments are designed to evaluate the predictive performance for drugs and cancer types on which the model was not previously trained.


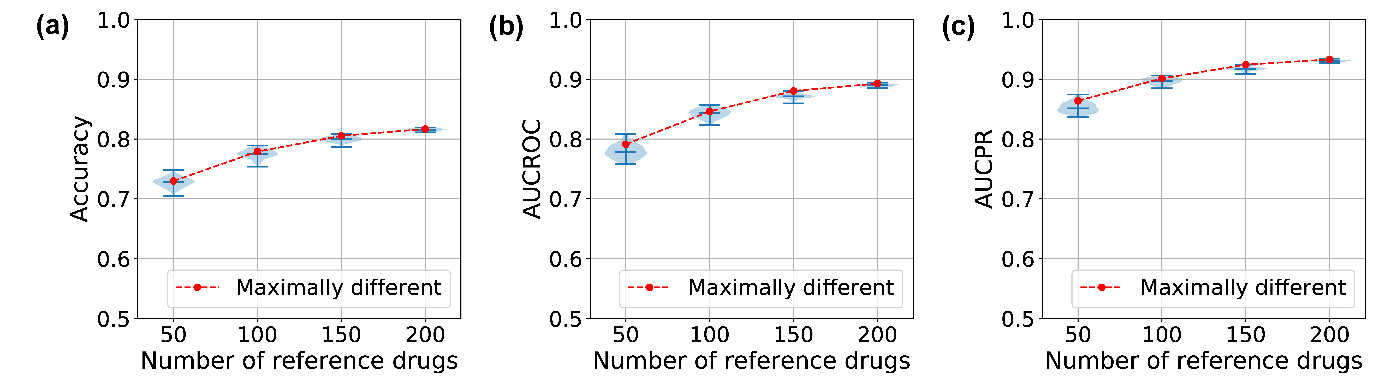


**Supplementary Fig. S3. Relationship between the predictive power and the number of reference drugs.** (a) Accuracy per number of reference drugs, (b) AUCROC, and (c) AUCPR; For each number of reference drugs, we randomized 100 times a subset of a given number of reference drugs from 222 drugs in GDSC dataset and made violin plots for the distributions of three metrics. Among the 100 subsets, a special subset of drugs with maximally different molecular structures from each other was marked to verify that the structural diversity of reference drugs affects the performance of RefDNN. The maximally different set was selected by the largest sum of pairwise Tanimoto coefficient values.
